# Overexpression, crystallization and preliminary X-­ray crystallographic analysis of the C-terminal cytosolic domain of mouse anoctamin 1

**DOI:** 10.1107/S1744309111027989

**Published:** 2011-09-29

**Authors:** Sang Ho Park, Ho Kyung Chung, Do Jin Kim, Mi Ra Han, Mi Seul Park, Uhtaek Oh, Hyun-Jung Kim, Byung Woo Han

**Affiliations:** a Research Institute of Pharmaceutical Sciences, College of Pharmacy, Seoul National University, Seoul 151-742, Republic of Korea; b Department of Chemistry, College of Natural Sciences, Seoul National University, Seoul 151-742, Republic of Korea; c College of Pharmacy, Chung-Ang University, Seoul 156-756, Republic of Korea

**Keywords:** anoctamin 1, transmembrane protein 16A, chloride channels

## Abstract

Transmembrane protein 16A (TMEM16A, also known as anoctamin 1; ANO1) is a bona fide Ca^2+^-activated chloride channel that is activated by intracellular Ca^2+^- and Ca^2+^-mobilizing stimuli and plays important roles in a variety of physiological functions. To elucidate the structural features of ANO1, structural analysis of the C-terminal cytosolic domain of mouse ANO1 (mANO1-CTD) was initiated. mANO1-CTD was overexpressed in *Escherichia coli* and was crystallized at 297 K using a reservoir solution consisting of 0.2 *M* sodium acetate trihydrate, 0.1 *M* Tris–HCl pH 8.5 and 30%(*w*/*v*) PEG 4000. X-­ray diffraction data were collected to 2.3 Å resolution. The crystals belonged to the orthorhombic space group *P*2_1_2_1_2_1_, with unit-cell parameters *a* = 73.96, *b* = 103.73, *c* = 114.71 Å. If it is assumed that eight copies of a monomer molecule are present in the crystallographic asymmetric unit, the crystal volume per protein mass (*V*_M_) is 2.38 Å^3^ Da^−1^ and the solvent content is 48.38%. Attempts to solve the structure of mANO1-CTD by the MAD method using seleno­methionine-labelled mANO1-CTD or heavy-atom-derivatized crystals are in progress.

## Abstract 2

The C-terminal cytosolic domain of mouse anoctamin 1 (mANO1, also known as TMEM16A) was cloned, overexpressed, purified and crystallized. The crystals belonged to space group *P*2_1_2_1_2_1_ and diffracted to 2.3 Å resolution.

## 1. Introduction

Ca^2+^-activated chloride channels (CaCCs) play essential roles in many physiological processes, including transepithelial secretion, cardiac and neuronal excitation, sensory transduction, smooth muscle contraction and fertilization (Eggermont, 2004 ▶; Frings *et al.*, 2000 ▶; Hartzell *et al.*, 2005 ▶; Large & Wang, 1996 ▶). Anoctamin 1 (ANO1) is a recently discovered member of the CaCCs which is highly expressed in secretory epithelial tissues, including ductal glands, superficial epithelia of the airway, and oviduct, where it has been implicated to play a key role in calcium-dependent chloride secretion (Caputo *et al.*, 2008 ▶; Schroeder *et al.*, 2008 ▶; Yang *et al.*, 2008 ▶).

ANO1 is one of the members of the anoctamin (ANO, also known as transmembrane protein 16; TMEM16) family of membrane proteins, which consists of ten members (ANO1–10) in mammals. ANO1 consists of 26 exons and has been predicted to code for a variety of proteins (Caputo *et al.*, 2008 ▶). It belongs to a protein family with eight transmembrane helices and N- and C-terminal domains that face the cytoplasm (Galindo & Vacquier, 2005 ▶). ANO1 is essential for Ca^2+^-dependent Cl^−^ currents in airways, the large intestine, salivary glands, pancreatic glands and hepatocytes (Ousingsawat *et al.*, 2009 ▶; Rock *et al.*, 2009 ▶; Romanenko *et al.*, 2010 ▶). Severe transport defects have been detected in epithelial tissues of ANO1-knockout mice, leading to reduced saliva production and attenuated mucociliary clearance of the airways (Lee & Foskett, 2010 ▶; Ousingsawat *et al.*, 2009 ▶; Rock *et al.*, 2009 ▶). Disruption of mouse ANO1 caused abnormal development of the trachea, indicating that the gene is a regulator of epithelial and smooth muscle cell organization in murine development (Rock *et al.*, 2009 ▶).

In addition to these results, recent studies have suggested the possibility that ANO1 proteins participate in tumourigenesis (Lee & Foskett, 2010 ▶; Yang *et al.*, 2008 ▶). The relationship between ANO1 and tumourigenesis has not been explained clearly, but a secretory environment might be important for tumour-cell proliferation. Thus, analysis of the ANO1 expression pattern in tumours could be used for the prediction or treatment of cancer.

**Figure 1:**
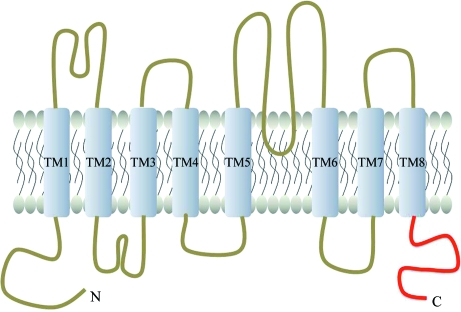
Predicted topology of mANO1. The cytosolic C-terminal domain of mANO1 is coloured red.

To facilitate the structural characterization of ANO1, we initiated structural study of the C-terminal cytosolic domain of mouse ANO1 (mANO1-CTD; residues 883–960; Fig. 1 ▶). In this report, we describe the overexpression, purification, crystallization and preliminary X-­ray crystallographic analysis of recombinant mANO1-CTD.

## 2. Methods and results

### 2.1. Protein expression and purification

mANO1-CTD was cloned into the expression vector pET-15b(+) (Novagen), adding a hexahistidine-containing 20-residue tag to the N-terminus. The recombinant protein was overexpressed in *Escherichia coli* Rosetta2(DE3)pLysS cells using Luria broth culture medium. Protein expression was induced by 0.5 m*M* isopropyl β-d-1-thiogalactopyranoside and the cells were incubated for 16 h at 293 K following growth to mid-log phase at 310 K. The cells were lysed by sonication in lysis buffer (20 m*M* Tris–HCl pH 7.5, 500 m*M* NaCl, 35 m*M* imidazole and 1 m*M* phenylmethanesulfonylfluoride). The supernatant was applied onto a HiTrap Chelating HP column (GE Healthcare) which was previously equilibrated with buffer *A* (20 m*M* Tris–HCl pH 7.5, 500 m*M* NaCl and 35 m*M* imidazole). The protein was eluted with a linear gradient of 0.035–1.0 *M* imidazole in buffer *A*. The eluted sample was further purified by gel filtration on a HiLoad 16/60 Superdex 200 prep-grade column (GE Healthcare) which was equilibrated with 20 m*M* Tris–HCl pH 7.5 and 200 m*M* NaCl. The buffer of the fractions containing mANO1-CTD was gradually changed to buffer *B* (20 m*M* Tris–HCl pH 7.5 and 50 m*M* NaCl) using a Amicon Ultra-15 centrifugal filter device (Millipore). The sample was applied onto a HiTrap SP ion-exchange column (GE Healthcare) which was previously equilibrated with buffer *B*. The protein was eluted with a linear gradient of 0.05−1.0 *M* NaCl in buffer *B*. The homogeneity of the purified protein was assessed by SDS–PAGE. The buffer of the fractions containing mANO1-CTD was changed to 20 m*M* Tris–HCl pH 7.5 and 100 m*M* NaCl to reduce the concentration of NaCl and purified mANO1-CTD was concentrated to a final concentration of 9 mg ml^−1^ using an Amicon Ultra-15 centrifugal filter device (Millipore).

### 2.2. Crystallization and X-ray data collection

**Figure 2:**
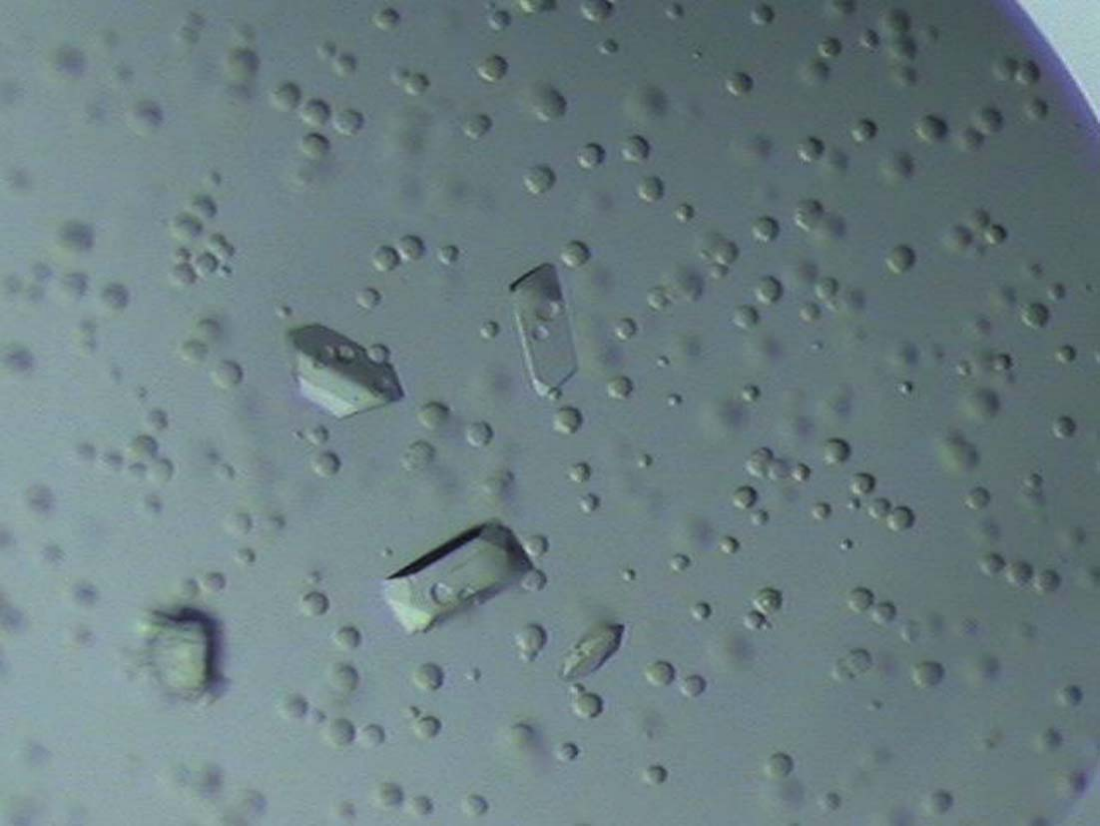
Native crystals of mANO1-CTD. The crystal dimensions are approximately 0.1 × 0.1 × 0.3 mm.

Crystals were grown by the sitting-drop vapour-diffusion method at 297 K by mixing equal volumes (1 µl each) of protein solution and reservoir solution. Crystals appeared after three weeks using a reservoir solution consisting of 0.1 *M* Tris–HCl pH 8.5, 0.2 *M* sodium acetate trihydrate and 30%(*v*/*v*) PEG 4000 with approximate dimensions of 0.1 × 0.1 × 0.3 mm (Fig. 2 ▶).

**Figure 3:**
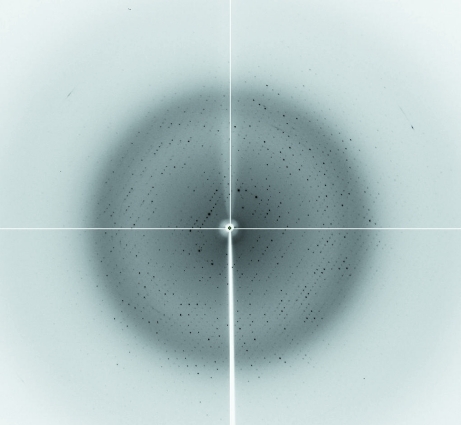
X-ray diffraction image from an mANO1-CTD crystal. The edge of the detector corresponds to a resolution of 2.0 Å.

For diffraction data collection, the crystals were directly soaked in a cryoprotectant solution composed of 30%(*v*/*v*) glycerol added to the reservoir solution. X-ray diffraction data were collected at 100 K on a Quantum 4R CCD detector (Area Detector Systems Corporation, Poway, California, USA) at the BL-6C experimental station of Pohang Light Source, Republic of Korea (Fig. 3 ▶). For each image the crystal was rotated by 1° and the raw data were processed using the *HKL*-2000 program suite (Otwinowski & Minor, 1997 ▶). A total of 280 647 measured reflections were merged into 40 051 unique reflections with an *R*_merge_ of 12.7% and a completeness of 99.9%. The space group was determined to be *P*2_1_2_1_2_1_ on the basis of systematic absences and symmetry of diffraction intensities using the *POINTLESS* program from *CCP*4 (Winn *et al.*, 2011 ▶). The unit-cell parameters are *a* = 73.96, *b* = 103.73, *c* = 114.71 Å. Table 1 ▶ summarizes the statistics of data collection. If it is assumed that eight copies of a monomer molecule are present in the crystallographic asymmetric unit, the crystal volume per protein mass (*V*_M_) is 2.38 Å^3^ Da^−1^ and the solvent content is 48.38%. Attempts to solve the structure of mANO1-CTD by the MAD method using selenomethionine-labelled mANO1-CTD or heavy-atom-derivatized crystals are in progress.

## Figures and Tables

**Table 1:** Data-collection statistics Values in parentheses are for the highest resolution shell.

X-ray source	Pohang Light Source beamline BL-6C
X-ray wavelength (Å)	1.23985
Temperature (K)	100
Space group	*P*2_1_2_1_2_1_
Unit-cell parameters (Å)	*a* = 73.96, *b* = 103.73, *c* = 114.71
Resolution range (Å)	20–2.30 (2.34–2.30)
Total/unique reflections	280647/40051
*R*_merge_† (%)	12.7 (45.9)
Data completeness (%)	100.0 (99.9)
Multiplicity	7.0 (6.5)
Average *I*/σ(*I*)	18.8 (3.8)

† *R*_merge_ = 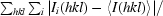 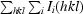, where *I*_*i*_(*hkl*) is the intensity of the *i*th measurement of reflection *hkl* and 〈*I*(*hkl*)〉 is the mean value of *I*_*i*_(*hkl*) for all *i* measurements.
